# Optic Neuritis as an Isolated Presentation of Kikuchi–Fujimoto Disease in a Pediatric Patient

**DOI:** 10.4274/balkanmedj.galenos.2019.2019.11.88

**Published:** 2020-04-10

**Authors:** Aydan Arslan, Courtney Lynn Kraus, Izlem Izbudak

**Affiliations:** 1 Department of Radiology, Johns Hopkins University School of Medicine, Baltimore, USA; 2 Department of Ophthalmology, Johns Hopkins University School of Medicine, Baltimore, USA

To the Editor,

Kikuchi–Fujimoto disease (KFD), which is also known as histiocytic necrotizing lymphadenitis, is a rare, idiopathic, and benign disease. It is self-limiting and predominantly characterized by cervical lymph node enlargement in young women, especially those of Asian descent (1,2).

A 12-year-old female patient was observed in the ophthalmology clinic with complaints of mild color deficiency, specifically red desaturation, in her right eye. The symptoms first began one month prior to the examination. Ophthalmology examination revealed that she had 20/20 vision in both eyes with no afferent pupillary defect (APD). Her color vision was diminished in the right eye (5/10 color plates in the right eye; 10/10 in the left). Visual fields were full to confrontation. Automated visual field testing was unreliable in both eyes, and it failed to reveal a focal deficit in the right eye.

Extraocular muscle motility was normal. Her external exam and anterior segments in both eyes were unremarkable. The funduscopic exam was notable for optic nerve pallor on the right. 

Laboratory examination showed elevated ESR/CRP levels, but other blood test values were within the normal ranges, as detailed in Table 1. Her past medical history and medications were unremarkable, except for persistent firm and intermittently painful submental and submandibular lymphadenopathy, which had been treated using multiple antibiotics. The year prior to her presentation to ophthalmology, she had undergone a submental lymph node biopsy, which resulted in the diagnosis of KFD. At the time of the presentation, she was not receiving any targeted treatment for this diagnosis.

Magnetic resonance imaging (MRI) of the brain and orbits with contrast showed a moderate asymmetric T2 hyperintense signal of the right optic nerve. Also, they indicated asymmetric enhancement and thickening of the orbital and intracanalicular segments of the right optic nerve compared with the left, compatible with optic neuritis (Figure 1). The brain parenchyma demonstrated normal signal intensity without the evidence of mass, hemorrhage, midline shift, or abnormal enhancement.

She was initiated on 20-mg oral prednisone administration daily that was tapered over three weeks. A follow-up MRI four months later with contrast revealed minimally asymmetric enhancement of the right optic nerve substantially decreased compared with the prior study. A minimally asymmetric T2 hyperintense signal of the right optic nerve was also noted (Figure 1). Her symptoms decreased but had not resolved at the time of the MRI. Therefore, her ophthalmologist decided to administer 375-mg/m^2^ rituximab for four weeks.

A follow-up ophthalmology exam after one year demonstrated a complete resolution with the same color vision in both eyes. Written informed consent was obtained from the patient’s parents.

KFD is an entity that is very rarely reported with ocular manifestations (only 22 patients, including our case) in literature. In summary, ocular manifestations of KFD were uveitis, retinal vein vasculitis, optic neuritis (2 patients, including our patient), lacrimal gland involvement, oculomotor palsy, papillary conjunctivitis, eyelid edema, periorbital edema, subretinal macular infiltrate, conjunctival injection, choroidal edema, papillary edema, and intraconal lesions of the apex. Herein we present a 12-year-old female patient with KFD and unilateral optic neuritis.

Optic neuritis is an acute inflammation of the optic nerve and one of the common causes of optic neuropathy. The causes of optic neuritis can be divided into infectious and noninfectious categories. The noninfectious category includes multiple sclerosis (MS), acute disseminated encephalomyelitis (ADEM), neuromyelitis optica (NMO), other systemic disorders such as lupus, or sarcoidosis. Exposure to toxins and radiation can also cause optic neuropathy (3,4).

In our patient, there were no findings of central nervous system demyelinating disease on MRI, including MS, ADEM, or NMO. The brain MRI was normal. Her serum NMO-IgG test was negative. Her clinical presentation, clinical examination, and laboratory tests did not support the diagnosis of rheumatologic or systemic diseases, such as SLE, sarcoidosis. There was no prior history of radiation treatment. The negative serodiagnostic tests helped to rule out HSV, HIV, and VZV. Therefore, her immunosuppressive treatment responsive optic neuritis was believed to be associated with KFD.

When evaluated together with the other optic neuritis case in the literature, KFD-induced lymphadenopathy and optic neuritis findings were at different times in both cases. While this was one year in our case, it was simultaneous in the previous case. In addition, the previous patient spontaneously recovered in four weeks. We used rituximab, and systemic steroid therapy, and our patient recovered after one year (2).

In conclusion, clinicians should be aware that KFD may be associated with ocular findings, such as optic neuritis. The follow-up of patients with KFD who have ocular involvement is recommended for potential recurrence or long-term damage.

## Figures and Tables

**Table 1:**
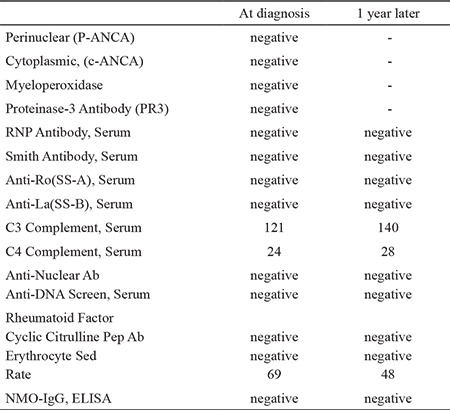
Blood test values of patient

**Figure 1:**
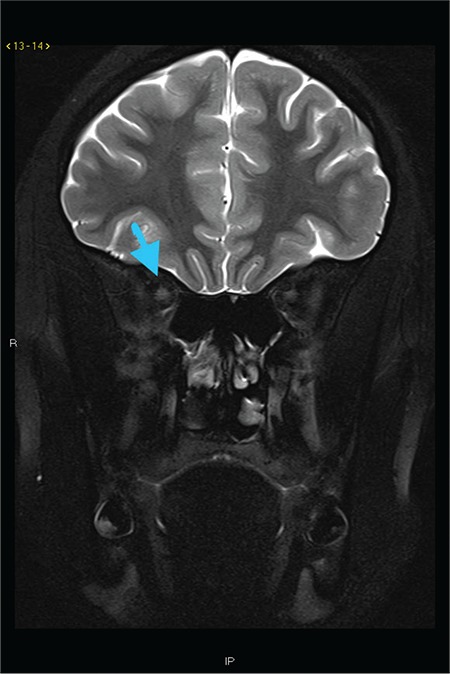
A 12-year-old female with Kikuchi–Fujimoto Disease. Subtraction post contrast T1 weighted coronal image showed asymmetric, abnormal enhancement in the right optic nerve (arrow).
